# Impact of averaging fNIRS regional coherence data when monitoring people with long term post-concussion symptoms

**DOI:** 10.1117/1.NPh.10.3.035005

**Published:** 2023-07-04

**Authors:** Ibukunoluwa K. Oni, Andrew P. Lapointe, Bradley G. Goodyear, Chantel T. Debert, Jeff F. Dunn

**Affiliations:** a University of Calgary, Department of Biomedical Engineering, Calgary, Alberta, Canada; b University of Calgary, Department of Radiology, Faculty of Medicine, Calgary, Alberta, Canada; c University of Calgary, Hotchkiss Brain Institute, Calgary, Alberta, Canada; d University of Calgary, Cumming School of Medicine, Experimental Imaging Centre, Calgary, Alberta, Canada; e University of Calgary, Cumming School of Medicine, Department of Clinical Neurosciences, Calgary, Alberta, Canada

**Keywords:** optics, photonics, light, functional near-infrared spectroscopy, concussion, functional near-infrared spectroscopy, mTBI

## Abstract

**Significance:**

Functional near-infrared spectroscopy (fNIRS), with its measure of delta hemoglobin concentration, has shown promise as a monitoring tool for the functional assessment of neurological disorders and brain injury. Analysis of fNIRS data often involves averaging data from several channel pairs in a region. Although this greatly reduces the processing time, it is uncertain how it affects the ability to detect changes post injury.

**Aim:**

We aimed to determine how averaging data within regions impacts the ability to differentiate between post-concussion and healthy controls.

**Approach:**

We compared interhemispheric coherence data from 16 channel pairs across the left and right dorsolateral prefrontal cortex during a task and a rest period. We compared the statistical power for differentiating groups that was obtained when undertaking no averaging, vs. averaging data from 2, 4, or 8 source detector pairs.

**Results:**

Coherence was significantly reduced in the concussion group compared with controls when no averaging was undertaken. Averaging all 8 channel pairs before undertaking the coherence analysis resulted in no group differences.

**Conclusions:**

Averaging between fiber pairs may eliminate the ability to detect group differences. It is proposed that even adjacent fiber pairs may have unique information, so averaging must be done with caution when monitoring brain disorders or injury.

## 1 Introduction

Functional near-infrared spectroscopy (fNIRS) is a technology that is increasingly being applied to monitor brain function in healthy, diseased, or injured brains.^1^^–^^6^ As it is portable and relatively accessible in terms of data collection and processing, and it may rival MRI for assessing brain function in a wide range of neurological conditions including concussion. Concussion is a mild traumatic brain injury (mTBI) that results in altered neurological states leading to symptoms of memory and perception deficits, headaches, and other behavioral changes.^7^^–^^10^ The functional nature of these symptoms makes fNIRS an ideal method for monitoring the injury. In this paper, we investigate fNIRS as a tool for assessing concussion.

Using fNIRS, we can detect changes in hemoglobin concentrations (oxy- and deoxy-hemoglobin). There are said to be several frequencies/oscillations contained within this signal that can be related to hemodynamics and metabolism in the brain.^11^^,^^12^ The synchronization of these oscillations plays a part in normal brain function and can be altered following injury to the brain. Wavelet coherence analysis has been used as a method to measure the synchronization between brain regions.^13^ One method of comparing these frequencies between brain regions is through wavelet coherence analysis. Wavelet coherence is calculated between two or more input time series signals. First, a wavelet transform is utilized to decompose each time series into a time-frequency signal with various frequency components, which in fNIRS are related to various physiological processes. The time-frequency signals are then compared with each other to determine coherence.^13^ We have shown previously that, when comparing healthy controls with patients with long term symptoms after a concussion, interhemispheric coherence is reduced in both pediatric and adult populations.^14^^,^^15^ Another study in hemiplegic stroke patients found increased coherence in right and left hemiplegic patients when compared with controls.^16^ Yet another study found that participants with major depression disorder had reduced coherence compared with the control group.^17^ Even studies that focus on control groups are able to determine changes in brain function during a task using this analysis technique.^18^^,^^19^ These studies show promise for the use of fNIRS and signal coherence analysis as a tool for assessing changes in control groups during a task and in neurological conditions such as concussion.

In coherence or magnitude analysis, averaging techniques are often applied to the data from different source detector pairs before quantifying changes in coherence or magnitude.^18^^–^^20^ This has the advantage of reducing outlying or spurious data points and making the analysis less susceptible to a source–detector pair with low signal and poor data. Thus, averaging reduces variation. Averaging data from different source–detector pairs before undertaking coherence analysis will also greatly reduce the time and effort involved in post-processing and analysis. The disadvantage of averaging is that one loses spatial information.

Previous literature on concussion, using either fNIRS^14^^,^^15^ or EEG,^21^^,^^22^ has shown that the dorsolateral prefrontal cortex (DLPFC) may be impaired after a concussion. For this reason, we chose to study the impact of data averaging in the DLPFC.

We aimed to explore two objectives. First, we wanted to see if the observation that fNIRS coherence was reduced in adults with long term symptoms after a concussion could be reproduced. This is important for validating fNIRS as a tool that can provide reproducible results and for strengthening the conclusion that functional impairment can still exist months after a concussion. Second, we aimed to show how grouping or averaging data from different source–detector pairs impacts the statistical power for detecting such impairments.

## 2 Methods

### 2.1 Participant Demographics

This study was approved by the Conjoint Health Research Ethics Board of the University of Calgary. Two groups of participants were recruited for this study. The control group included 30 participants (15M, 15F, age: 35.77±14.77 years) who had not experienced a concussion in at least the past year. The concussion group included 15 participants (4M, 11F, age: 42.93±15.04 years, time since injury: 2.6±0.91 months). Concussion participants were recruited from an outpatient brain injury clinic at an academic hospital seen from June 2020 to March 2022. They were referred to this brain injury clinic by practitioners in the community for treatment of persistent post-concussive symptoms (PPCS); therefore, this concussion sample is highly selective. Concussion was diagnosed by a medical practitioner based on the definition from the American Congress of Rehabilitation Medicine (ACRM).^7^ PPCS was diagnosed by a specialist using the ICD-10 criteria.^23^ All concussion participants included in this study were confirmed to have experienced a concussion within the last 6 months (Table 1). Participants were included in the study if they were within 6 months of their concussion diagnosis, were currently experiencing PPCS after their injury, were between the ages of 18 and 65 years, had no pre-existing neurological disorders, and currently were not using psychoactive drugs or medication.

**Table 1:** Demographics of PPCS participants included in the study.

Age	Sex	Symptom score	Prior mTBI	Time post-injury (months)
63	M	29	4	3
42	F	36	4	3
24	M	40	6	3
42	F	47	5	3
53	M	46	47	3
57	F	14	1	3
25	M	21	14	1
52	F	45	11	3
34	F	25	13	3
63	F	35	14	3
53	F	2	7	4
57	F	57	6	3
21	F	42	7	1
43	F	46	2	2
25	F	52	12	1

### 2.2 Data Acquisition

We acquired fNIRS deoxygenated (Hb) and oxygenated (HbO) hemoglobin data using the NIRx NIRScoutX (Berlin, Germany) at a sampling rate of 3.90625 Hz. The fNIRS optodes (sources and detectors) were placed ∼30 to 40 mm apart to maximize the signal obtained from the brain.^24^ The optodes were placed on the scalp according to the EEG 10-20/10-5 system.^25^^,^^26^ This allowed for accurate placement of the fNIRS channels on the scalp to cover the DLPFC for functional brain mapping.^26^ Another set of detectors were placed ∼8  mm from the source optodes to form the short distance channels. The optodes were arranged as shown in Fig. 2. Fiber positions on the DLPFC were estimated using the fNIRS optodes’ location decider v2.2 with the specificity threshold set at “30%.”^27^ fNIRS optodes were calibrated to ensure a good signal quality using the calibration feature on the NIRx NIRStar software.

### 2.3 Task

**Fig. 1:**
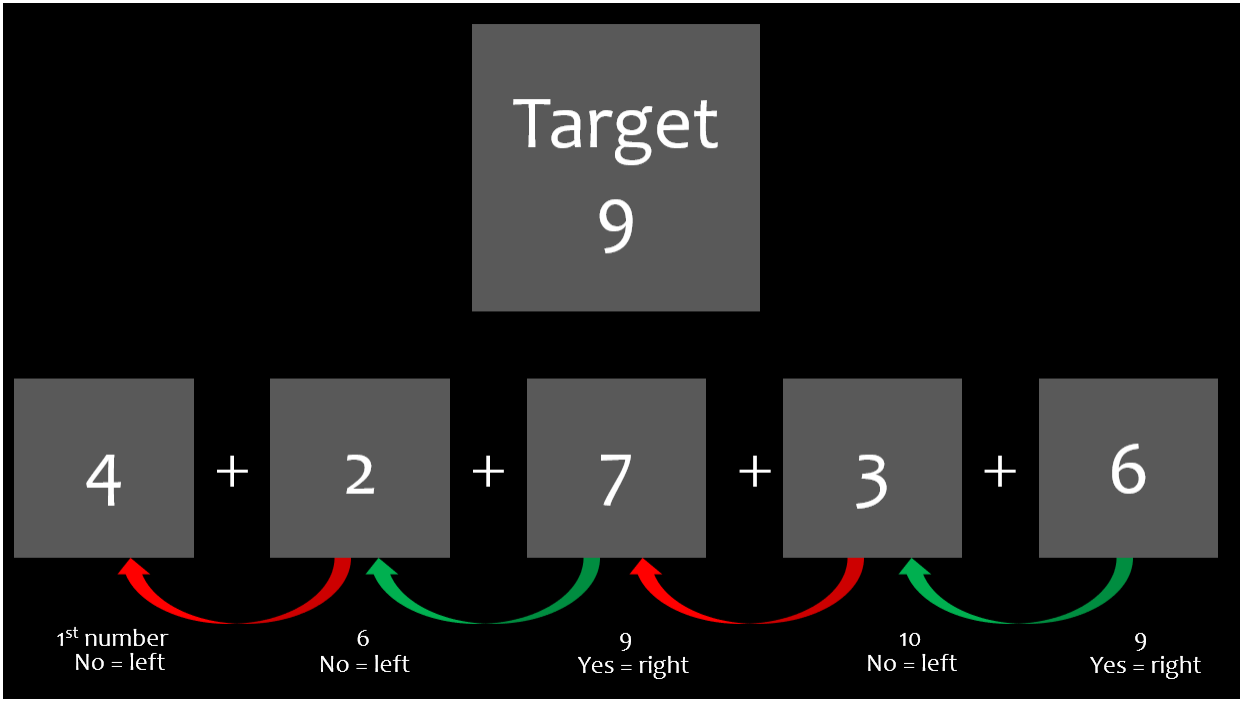
Example of the PVSAT. The participant was given a target number and was asked to add numbers and respond by tapping an arrow key if the numbers added to the target or not.

A resting state task and a paced visual serial addition task^28^ (PVSAT) were completed for this study. Participants were trained on the task before data collection began. Participants were seated ∼75 cm from a screen that projected images to identify the task to be completed. First, they completed an 8 min rest period with the participant being asked to focus on a cross in the middle of the screen. Next, they completed the PVSAT (Fig. 1). The PVSAT was presented in a block design (12 blocks). Participants were given a target number (9, 10, or 11) before each task block. For each block, they were presented with a single digit number on the screen that was replaced at an inter-stimulus interval of 1 s. Participants were asked to add the number currently presented on the screen to the number previously presented. If the two numbers add to the target number, they were asked to press the “right” arrow key on the keyboard. If they do not add to the target number, they were asked to press the “left” arrow key.

### 2.4 Data Analysis

Intensity data were processed using the Homer2 software package^29^ in MATLAB (The Mathworks, Natick, Massachusetts) and following the steps. The intensity data recorded via the NIRStar software were converted into the Homer2 “.nirs” format to be further processed. The intensity data extracted from the “.nirs” file were converted to delta optical density. Movement related artifacts were removed from the data using the Homer2 spline motion correction algorithm^29^ with a Savitzky–Golay smoothing filter. Optical density data were then converted to hemoglobin concentration using the modified Beer–Lambert law^29^^,^^30^ and the age-dependent differential pathlength factor,^31^ and bandpass filtering (0.001 to 1.9 Hz) was performed. Next, we performed the physiological regression of the short channels.^32^^,^^33^ This was done using the equation posited by Saager and Berger with the replacement of the alpha value with the value of the correlation between the long and short channel time series. Finally, we calculated interhemispheric coherence on the time series output of the short channel regression using the MATLAB wavelet coherence function. This function uses a Morlet wavelet as its basis for the coherence calculation with a moving average filter to smooth across time and frequency. Although the coherence calculation was done on the whole frequency band (0.001 to 1.9 Hz), we extracted the coherence values between 0.01 and 0.06 Hz for further analysis.

**Fig. 2:**
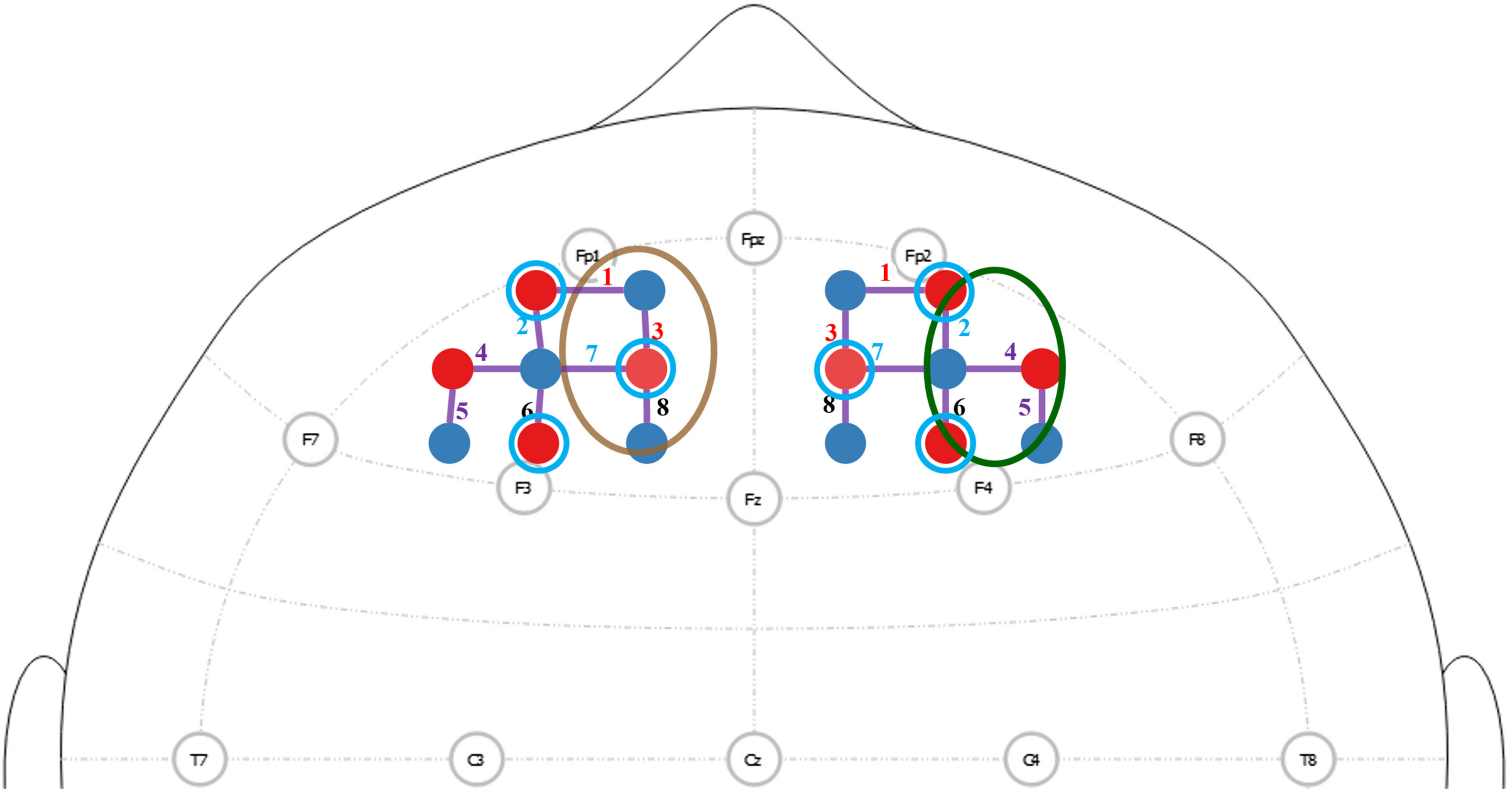
fNIRS channel locations in the DLPFC. Sources are shown in red and detectors in blue with the lines between them representing the channels. Short channels are indicated with the light blue circle surrounding the source optode. The channels are numbered and color-coded to represent the channels on either hemisphere selected for the coherence calculation when no averaging and when two channels were averaged together. The color-coded ovals represent the four channels averaged together on each hemisphere.

To study the impact of averaging the interhemispheric coherence data from different channel pairs (colored numbers in Fig. 2), we obtained data from channel pairs in the DLPFC, with eight source–detector pairs in each of the left and right DLPFC. We undertook coherence analysis in which no channel pairs were averaged, resulting in eight coherence values from eight source–detector pairs. We applied the same analysis to the data averaged from two nearest pairs, resulting in four coherence values per person (colored lines in Fig. 2); four nearest pairs, resulting in two coherence values per person (colored ovals in Fig. 2); and all eight pairs, resulting in one coherence value per person.

### 2.5 Statistical Analysis

A linear mixed model was used to assess group differences (control versus concussion) based on the coherence value with participant “id” listed as a random factor.^33^ Model assumptions were verified using the “performance” package^34^ in RStudio (version 4.2.0),^35^ which reports the model fit, collinearity, homogeneity of variance, and normality of residual and random effects. To alleviate bias due to an unbalanced dataset, we performed a permutation test (n=1000) on all variations of signal combinations and compared the model statistics.

The most common metric to interpret statistical models is the p-value,^36^ which provides insight into whether an effect is present or, alternatively stated, whether it is “statistically significant.”^37^ It provides insight into whether there is a difference between two groups, in our case controls and concussion. The effect size is suggested to be accompanied alongside the p-values to allow for interpretation of the strength between variables, which provides insight into the magnitude of the effect.^37^^–^^39^ The magnitude of effect sizes is labeled, either small ($\eta^{2}=0.0099$), medium ($\eta^{2}=0.0588$), or large ($\eta^{2}=0.1379$).^39^^,^^40^ Effect sizes that are considered “small” may still be important as variances from unmeasured variables may have decreased what might have been a medium or large effect.^40^ It is expected that a p-value less than the threshold for statistical significance (p<0.05) coupled with a medium effect size would give confidence that the concussion group could be differentiated from the controls.

To determine the impact of averaging, we used both the p-value and effect size as criteria when comparing group (control versus concussion) differences. The significance of the model effects was evaluated with the Satterthwaite approximation for degrees of freedom, an alpha level of 0.05 was used for all statistical tests. Descriptive statistics data were calculated to show the mean, standard deviation (SD), coefficient of variation (CV), and maximum coherence value for each channel pair and group. All statistical analyses were performed in RStudio (version 4.2.0).^35^

## 3 Results

We applied different averaging strategies to the hemodynamic data (e.g., HbO and Hb) during both PVSAT and resting state. We examined which averaging strategy improved the discrimination between the control and concussion groups based on their effect size and p-values. Hb did not show any differences in interhemispheric coherence between the groups during both PVSAT and resting state; therefore, only HbO is explored/reported in this paper.

Table 2 shows the interhemispheric coherence during PVSAT, between similar channel pairs when they are averaged/not averaged before calculating coherence. When no channel pairs are averaged before calculating coherence, the mean coherence ranged from 0.36 to 0.51 in controls and 0.35 to 0.44 in concussed participants, and the maximum values ranged from 0.54 to 0.82 in controls and 0.43 to 0.65 in concussed participants. The CVs ranged from 0.22 to 0.33 in controls and 0.18 to 0.29 in concussed participants. When all eight channel pairs were averaged before calculating coherence, the coherence was 0.49 in controls and 0.45 in concussed participants with a CV of 0.3 to 0.28, respectively. Table 3 shows similar data collected during the resting state. The mean coherence ranged from 0.34 to 0.5 in controls and 0.31 to 0.44 in concussed participants, and the maximum values ranged from 0.53 to 0.83 in controls and 0.45 to 0.69 in concussed participants. The CVs ranged from 0.2 to 0.4 in controls and 0.18 to 0.35 in concussed participants. When all eight channel pairs were averaged before analysis, the coherence was 0.49 in controls and 0.46 in concussed participants with a CV of 0.25 to 0.3, respectively.

**Table 2:** Descriptive statistics of the HbO data during PVSAT. “Max” represents the maximum value for the interhemispheric coherence data between the channel pairs (Fig. 1). “SD of Max from mean” represents the number of SDs of the maximum coherence from the mean coherence. The “Mean ± SD” and “CV” represent the mean, SD, and CV for the HbO interhemispheric coherence during PVSAT. “Channel pair” represents the left- and right-side locations (Fig. 1) of the coherence value calculations.

Averaging	Channel pair	Group	CV	Max	SD of Max from mean	Mean ± SD
None	1	Control	0.25	0.78	2.25	0.51 ± 0.12
None	1	Concussion	0.29	0.65	1.62	0.44 ± 0.13
None	2	Control	0.25	0.81	2.75	0.48 ± 0.12
None	2	Concussion	0.18	0.54	1.50	0.42 ± 0.08
None	3	Control	0.26	0.75	2.91	0.43 ± 0.11
None	3	Concussion	0.23	0.54	2.25	0.36 ± 0.08
None	4	Control	0.28	0.81	3.17	0.43 ± 0.12
None	4	Concussion	0.20	0.51	2.00	0.37 ± 0.07
None	5	Control	0.23	0.54	2.25	0.36 ± 0.08
None	5	Concussion	0.21	0.53	2.13	0.36 ± 0.08
None	6	Control	0.33	0.82	2.71	0.44 ± 0.14
None	6	Concussion	0.25	0.58	1.90	0.39 ± 0.1
None	7	Control	0.22	0.56	2.13	0.39 ± 0.08
None	7	Concussion	0.23	0.60	2.22	0.4 ± 0.09
None	8	Control	0.24	0.60	2.33	0.39 ± 0.09
None	8	Concussion	0.18	0.43	1.33	0.35 ± 0.06
Two	1_3	Control	0.25	0.78	2.25	0.51 ± 0.12
Two	1_3	Concussion	0.29	0.65	1.62	0.44 ± 0.13
Two	2_7	Control	0.25	0.81	2.75	0.48 ± 0.12
Two	2_7	Concussion	0.18	0.54	1.50	0.42 ± 0.08
Two	4_5	Control	0.28	0.81	3.17	0.43 ± 0.12
Two	4_5	Concussion	0.20	0.51	2.00	0.37 ± 0.07
Two	6_8	Control	0.24	0.60	2.33	0.39 ± 0.09
Two	6_8	Concussion	0.18	0.43	1.33	0.35 ± 0.06
Four	1378	Control	0.26	0.84	3.08	0.47 ± 0.12
Four	1378	Concussion	0.19	0.55	1.63	0.42 ± 0.08
Four	2456	Control	0.27	0.79	2.67	0.47 ± 0.12
Four	2456	Concussion	0.23	0.54	1.30	0.41 ± 0.1
Eight	1-8	Control	0.30	0.79	2.00	0.49 ± 0.15
Eight	1-8	Concussion	0.28	0.65	1.67	0.45 ± 0.12

**Table 3:** Descriptive statistics of the HbO data during the resting state. “Max” represents the maximum value for the interhemispheric coherence data between the channel pairs (Fig. 1). “SD of Max from mean” represents the number of SDs of the maximum coherence from the mean coherence. The “Mean ± SD” and “CV” represent the mean, SD, and CV for the HbO interhemispheric coherence during the resting state. “Channel pair” represents the left- and right-side locations (Fig. 1) of the coherence value calculations.

Averaging	Channel pair	Group	CV	Max	SD of Max from mean	Mean ± SD
None	1	Control	0.29	0.75	1.79	0.5 ± 0.14
None	1	Concussion	0.27	0.57	1.55	0.4 ± 0.11
None	2	Control	0.25	0.71	2.55	0.43 ± 0.11
None	2	Concussion	0.24	0.55	1.40	0.41 ± 0.1
None	3	Control	0.30	0.70	2.58	0.39 ± 0.12
None	3	Concussion	0.26	0.55	1.80	0.37 ± 0.1
None	4	Control	0.32	0.72	2.31	0.42 ± 0.13
None	4	Concussion	0.20	0.55	1.88	0.4 ± 0.08
None	5	Control	0.20	0.53	2.71	0.34 ± 0.07
None	5	Concussion	0.18	0.45	2.33	0.31 ± 0.06
None	6	Control	0.40	0.83	2.41	0.42 ± 0.17
None	6	Concussion	0.35	0.69	1.67	0.44 ± 0.15
None	7	Control	0.24	0.59	2.44	0.37 ± 0.09
None	7	Concussion	0.29	0.56	1.55	0.39 ± 0.11
None	8	Control	0.30	0.62	1.83	0.4 ± 0.12
None	8	Concussion	0.23	0.52	2.25	0.34 ± 0.08
Two	1_3	Control	0.29	0.75	1.79	0.5 ± 0.14
Two	1_3	Concussion	0.27	0.57	1.55	0.4 ± 0.11
Two	2_7	Control	0.25	0.71	2.55	0.43 ± 0.11
Two	2_7	Concussion	0.24	0.55	1.40	0.41 ± 0.1
Two	4_5	Control	0.32	0.72	2.31	0.42 ± 0.13
Two	4_5	Concussion	0.20	0.55	1.88	0.4 ± 0.08
Two	6_8	Control	0.30	0.62	1.83	0.4 ± 0.12
Two	6_8	Concussion	0.23	0.52	2.25	0.34 ± 0.08
Four	1378	Control	0.23	0.63	2.00	0.43 ± 0.1
Four	1378	Concussion	0.22	0.53	1.33	0.41 ± 0.09
Four	2456	Control	0.26	0.72	2.64	0.43 ± 0.11
Four	2456	Concussion	0.22	0.52	1.44	0.39 ± 0.09
Eight	1-8	Control	0.25	0.78	2.42	0.49 ± 0.12
Eight	1-8	Concussion	0.30	0.72	1.86	0.46 ± 0.14

### 3.1 Channel Pair Differences

Preliminary analysis revealed that there were no groups by channel pair interactions either during PVSAT or the resting state. Statistical differences were noted between individual channel pairs with a medium effect size ($\eta^{2}>=0.058$)^39^^,^^40^ during PVSAT [F(7,280)=4.63, p<0.001, $\eta^{2}=0.1$] when no averaging was performed (Table 4) and [F(3,120)=3.33, p<0.05, $\eta^{2}=0.06$] when only two channel pairs were averaged. There was no significant difference between the channel pairs during PVSAT when observing the four channel averaged data [F(1,39)=0.25, p=0.62, $\eta^{2}<0.01$]. Differences during the resting state showed similar responses with a medium effect size [F(7,280)=5.54, p<0.001, $\eta^{2}=0.12$] when no averaging was performed and a large effect [F(3,120)=7.86, p<0.001, $\eta^{2}=0.14$] when only two channel pairs were averaged, but no effect when four channel pairs were averaged [F(1,40)=0.11, p=0.74, $\eta^{2}<0.01$].

**Table 4:** Difference between channel pairs during the resting state and PVSAT sorted by the effect size.

Averaging	Task	SS	DFn	DFd	F	p	Effect size
Two	PVSAT	0.22	3	120	7.86	< 0.001	0.14
None	PVSAT	0.35	7	280	5.54	< 0.001	0.12
None	Rest	0.40	7	280	4.63	< 0.001	0.10
Two	Rest	0.13	3	120	3.33	0.02	0.06
Four	PVSAT	0.00	1	40	0.11	0.74	0.00
Four	Rest	0.00	1	39	0.25	0.62	0.00

### 3.2 Channel Pair Averaging

We found statistical differences between groups when observing the different averaging strategies (Table 5). It was determined that averaging all eight fibers into one amplitude-time series before undertaking coherence analysis gives no significant difference between groups during PVSAT [F(1,43)=3.932, p=0.056, $\eta^{2}=0.084$] and resting state [F(1,43)=0.371, p=0.58, $\eta^{2}=0.009$], whereas averaging less channel pairs tends to improve the discrimination. During PVSAT, the group difference was significant with no averaging [F(1,43)=8.419, p=0.010, $\eta^{2}=0.164$] and when averaging two channel pairs [F(1,43)=6.435, p=0.023, $\eta^{2}=0.13$]. Averaging four channel pairs did not show a group difference [F(1,43)=3.202, p=0.079, $\eta^{2}=0.069$]. Resting state analysis did not show any group differences for no averaging [F(1,43)=3.743, p=0.062, $\eta^{2}=0.08$], when averaging two channel pairs [F(1,43)=0.64, p=0.438, $\eta^{2}=0.015$], and averaging four channel pairs [F(1,43)=0.067, p=0.802, $\eta^{2}=0.002$]. The effect size between averaging strategies during the resting state was small, ranging from (0.009 to 0.08), and medium to large during PVSAT (0.084 to 0.16). With resting state and PVSAT data, the effect size was largest with no averaging.

**Table 5:** Difference between groups (concussed and controls) during the resting state and PVSAT based on the permutation test (n=1000).

Averaging	Task	DFd	SS	F	p	Effect size
None	PVSAT	43	0.125	8.419	0.010	0.164
Two	PVSAT	43	0.063	6.435	0.023	0.130
Four	PVSAT	43	0.015	3.202	0.079	0.069
Eight	PVSAT	43	0.013	3.932	0.056	0.084
None	Rest	43	0.065	3.743	0.062	0.080
Two	Rest	43	0.007	0.640	0.438	0.015
Four	Rest	43	0.000	0.067	0.802	0.002
Eight	Rest	43	0.001	0.371	0.580	0.009

## 4 Discussion

We confirmed a reduction in fNIRS HbO coherence located in the DLPFC in adults with PPCS. This was observed in a prior study by our group.^15^ We also investigated the impact of averaging channel pair data on the statistical power of coherence analysis to detect group differences. We found that, when averaging a small number of channel pairs together (two or less), we are better able to statistically differentiate between groups than when averaging more channel pairs (four and eight channel pairs).

### 4.1 Group Differences Between Concussion and Controls

#### 4.1.1 Identifying region of interest for group difference calculation

Cognitive deficits (e.g., in attention or memory) are known to occur post-injury in PPCS patients. Deficits in working memory have been shown in patients with PPCS to be located within the frontal regions of the brain.^15^^,^^41^^–^^43^ fNIRS provides a great tool to observe the cognitive deficits that these patients experience as it has been shown to be sensitive to these types of changes.^15^^,^^43^ Therefore, we obtained data from channel pairs placed in this region (e.g., DLPFC) to identify the changes that occur after the injury. In this region, we observed if averaging channel pairs together changed their ability to detect differences between control and concussion participants.

#### 4.1.2 Impact of averaging on detecting group differences

We found that increasing the number of channel pairs averaged (above two channel pairs) decreased the ability to distinguish the concussion group from the controls (Table 5) as averaging both four and eight channel pairs was unable to detect group differences. This conclusion was supported using both p-values and effect sizes. The effect size was shown to be larger when either no channel pairs ($\eta^{2}=0.27$) or two channel pairs ($\eta^{2}=0.20$) compared with when all channel pairs were averaged together during PVSAT ($\eta^{2}=0.13$). The effect size for when four channel pairs were averaged was ($\eta^{2}=0.1$). The p-values also supported this conclusion. The p-value with data collected during PVSAT was p<0.01 for no channel pair averaging, p<0.05 for two channel pair averaging, p=0.09 for four channel pair averaging, and p=0.05 when averaging all channel pairs. As we average more channel pairs before calculating coherence, we lose the ability to discriminate between the patient group and controls. This would suggest that the use of a smaller number of channel pairs (which would also reduce data collection time) might be ideal for fNIRS studies once the relevant regions have been identified.

#### 4.1.3 Impact of task selection on detecting group differences

It remains unclear which task (e.g., resting state, finger-tapping) is best at differentiating concussion patients using interhemispheric coherence. Prior research in the field has shown group differences between controls and concussed populations using both an active task (e.g., PVSAT,^44^^,^^45^ finger tapping,^46^ visual shifting attention,^2^ and n-back^15^) and rest.^47^^,^^48^ In this study, we found that an active task discriminated groups better than a resting state task. However, this result was highly affected by the number of channel pairs that were averaged when drawing this group comparison. As such, our results indicate that the task choice may not be as vital as post-processing (i.e., averaging) decisions.

#### 4.1.4 Why is averaging reducing sensitivity?

In this study, we found that averaging more than two channel pairs had a negative effect on our sensitivity to detecting group differences. Because averaging is a method used to reduce variation within a dataset, a measure of variation within the data might prove useful in explaining why this is the case. A prior study stated that observed differences in averaged data are influenced by the amount of variation in the distributions.^49^ We evaluated the distribution of fNIRS data within each averaging method using measures of dispersion/variation. For our measures of variation within the data, we focused on the SD and CV. The SD examines the dispersion of a data set.^50^ This is helpful in determining the spread of the dataset. We found that the SD of the coherence data was consistent when no averaging was applied and when only two channel pairs were averaged. It subsequently reduced when averaging four and eight channel pairs. The next measure of dispersion, CV, measures the variation of SD from the mean.^51^ This provides a measure of dispersion of the data points around the mean value. We found that the CV was higher when more channel pairs were averaged, although the individual channel pairs had a wider range of CV. Both SD and CV were useful in describing the variation between participants and explaining the differences that we observe between the concussion and control groups.

We observed that, during PVSAT, we could discriminate the PPCS group from controls when using either no averaging or averaging of two channel pairs. Similar to previous work in our lab, we found improved discrimination during an active task compared with data collected during the resting state.^14^^,^^15^ These data indicate that, even in adjacent channel pairs, there could be unique hemodynamic information that would be lost with averaging.

This paper supports the premise that fNIRS can detect changes in the brain post-concussion. By analyzing the data on a group basis, our goal was to optimize collection and analysis protocols to improve concussion monitoring. By increasing sensitivity, this information could be used toward optimizing protocols for individualized medicine. Continued improvements in sensitivity to concussion are needed to achieve the goal of optimizing protocols for individual PPCS assessment of treatment response, progression, and injury severity.

## 5 Conclusion

In this study, we confirmed that interhemispheric coherence is reduced in an adult population with PPCS. This is an important reproducibility study as, to date, little imaging has been explored as a marker of injury in patients with PPCS. fNIRS is portable and inexpensive, and it provides important pathophysiological information to better understand the underpinnings of injury in patients with PPCS.

This study contributes to the existing body of knowledge on the effects of averaging on a dataset. With the demonstration of loss of spatial contrast due to averaging, we are extending the knowledge of the effect of averaging on a dataset to fNIRS analysis in concussion research.

This study explores important evaluations of fNIRS analysis that impacts the field of near infrared spectroscopy. We observed that averaging channel pairs from a region of interest influences the ability to differentiate between groups. We hypothesize that the individual channels, even adjacent channels, may have unique information. Therefore, averaging of channel pairs in fNIRS studies should be done with caution.

## Code, Data, and Materials Availability

Provision of the data and code used in this paper will be considered upon request.
